# Darbepoetin alfa for treating chemotherapy-induced anemia in patients with a baseline hemoglobin level < 10 g/dL versus ≥10 g/dL: an exploratory analysis from a randomized, double-blind, active-controlled trial

**DOI:** 10.1186/1471-2407-9-311

**Published:** 2009-09-03

**Authors:** Johan Vansteenkiste, Michael Hedenus, Pere Gascon, Carsten Bokemeyer, Heinz Ludwig, Jan Vermorken, Lisa Hamilton, Ken Bridges, Beatriz Pujol

**Affiliations:** 1 Respiratory Oncology Unit (Pulmonology), University Hospital Gasthuisberg, Herestraat 49, B-3000 Leuven, Belgium; 2 Department of Medicine, Sundsvall Hospital, Sundsvall, Sweden; 3 Department of Medical Oncology at the Hospital Clínic de Barcelona, University of Barcelona, Barcelona, Spain; 4 Department of Oncology (Hematology with Section Pneumology), University Hospital Hamburg Eppendorf, Hamburg, Germany; 5 1st Department of Medicine at the Center for Oncology and Haematology, Wilhelminenspital der Stadt Wien, Vienna, Austria; 6 Department of Medical Oncology, University Hospital Antwerp, Edegem, Belgium; 7 Biostatisics and Epidemiology, Amgen Ltd, Cambridge, UK; 8 Hematology/Oncology Therapeutic Area, Amgen Inc., Thousand Oaks, CA, USA; 9 Hematology/Oncology Therapeutic Area, Amgen Europe, Zug, Switzerland

## Abstract

**Background:**

Several studies have shown that darbepoetin alfa, an erythropoiesis-stimulating agent (ESA), can reduce transfusions and increase hemoglobin (Hb) levels in patients with chemotherapy-induced anemia (CIA). Recent safety concerns, however, have prompted changes to ESA product information. In the European Union and United States, ESA therapy initiation for CIA is now recommended at a Hb level ≤10 g/dL. The present exploratory analysis examined how ESA initiation at this Hb level may impact patient care.

**Methods:**

Data from a phase 3 randomized trial were retrospectively reanalyzed. CIA patients with nonmyeloid malignancies were randomized 1:1 to 500 mcg darbepoetin alfa every three weeks (Q3W) or 2.25 mcg/kg darbepoetin alfa weekly (QW) for 15 weeks. A previously published report from this trial showed Q3W dosing was non-inferior to QW dosing for reducing transfusions from week 5 to end-of-the-treatment period (EOTP). In the present analysis, outcomes were reanalyzed by baseline Hb <10 g/dL and ≥10 g/dL. Endpoints included transfusion rates, Hb outcomes, and safety profiles.

**Results:**

This study reanalyzed 351 and 354 patients who initiated ESA therapy at a baseline Hb of <10 g/dL or ≥10 g/dL, respectively. From week 5 to EOTP, the estimated Kaplan-Meier transfusion incidence (Q3W vs QW) was lower in the ≥10 g/dL baseline-Hb group (14% vs 21%) compared with the <10 g/dL baseline-Hb group (36% vs 41%). By week 5, the ≥10 g/dL baseline-Hb group, but not the <10 g/dL baseline-Hb group, achieved a mean Hb ≥11 g/dL. The Kaplan-Meier estimate of percentage of patients (Q3W vs QW) who achieved Hb ≥11 g/dL from week 1 to EOTP was 90% vs 85% in the ≥10 g/dL baseline-Hb group and 54% vs 57% in the <10 g/dL baseline-Hb group. Both baseline-Hb groups maintained mean Hb levels <12 g/dL and had similar safety profiles, though more patients in the ≥10 g/dL baseline-Hb group reached the threshold Hb of ≥13 g/dL.

**Conclusion:**

In this exploratory analysis, darbepoetin alfa Q3W and QW raised Hb levels and maintained mean Hb at <12 g/dL in both baseline-Hb groups. The ≥10 g/dL baseline-Hb group had fewer transfusions and faster anemia correction. Additional studies should prospectively evaluate the relationship between Hb levels at ESA initiation and outcomes.

**Trial Registration:**

ClinicalTrials.gov Identifier NCT00118638.

## Background

Patients with cancer who receive myelosuppressive chemotherapy may develop chemotherapy-induced anemia (CIA), which can be a debilitating condition resulting in increased morbidities and fatigue [1,2]. Erythropoiesis-stimulating agents (ESAs) are recombinant therapeutic agents (such as darbepoetin alfa and Epoetin alfa) that are used to treat anemia. Another method used to treat anemia is red blood cell (RBC) transfusions, which have only a transient effect and are associated with risks, such as exposure to infectious agents [3] and the more common risk of transfusion-related acute lung injury [4]. In addition, a retrospective cohort study recently observed that transfusions were associated with an increased incidence of thromboembolic events and increased mortality in hospitalized cancer patients [5]. There are also safety concerns with ESAs, including the established increased risk of venous thromboembolic events [6]. However, ESAs are currently the only therapeutic alternative to transfusions.

Numerous clinical trials have demonstrated the efficacy of ESAs for reducing the incidence of transfusions and raising hemoglobin (Hb) levels in cancer patients receiving chemotherapy [7-12]. Some studies have also indicated that ESAs can reduce fatigue in this patient population [2,10,13,14], as recognized by evidence-based guidelines [15]. ESAs are currently approved in both the European Union (EU) and the United States (US) for treating anemia in cancer patients receiving myelosuppressive chemotherapy [16-19].

Though ESAs appear to have an established efficacy profile and favorable adverse-event profile in adult cancer patients, recent findings from several individual studies conducted in off-label ESA settings have raised concerns regarding the potential for ESA-associated adverse survival and disease progression outcomes [8,20-26]. Information from eight individual studies reporting increased mortality and/or disease progression with ESA use have been added globally to all ESA product information [16-19]. Although 4 of these 8 studies were conducted in the approved chemotherapy setting, 3 of the 4 were anemia-prevention studies that allowed inclusion of patients with high baseline Hb levels [20,22,25] and all 4 studies targeted high Hb values (≥12 g/dL) [8,20,22,25]. The 4 studies not conducted in the chemotherapy setting were performed in off-label settings, such as patients not receiving chemotherapy or radiotherapy (ie, anemia of cancer [AoC]) [24,26] and patients receiving radiotherapy only [21,23].

In addition to describing information from these studies, the EU product information for all ESAs [16,17] also states that 1) ESA therapy should be initiated at a Hb level ≤10 g/dL, 2) the Hb target range should be 10 to 12 g/dL, and 3) a sustained Hb level > 12 g/dL attained with ESA use should be avoided. The US ESA product information [18,19] recommends that ESAs 1) not be used in the AoC or radiotherapy alone settings, 2) are not indicated for the treatment of anemia in the chemotherapy setting when the anticipated outcome is cancer cure, 3) be initiated in patients with Hb levels < 10 g/dL, and 4) be used to maintain the lowest Hb level sufficient to avoid transfusions.

Meta-analyses have been performed to examine the body of evidence available from controlled ESA studies performed in cancer patients. Fifty-nine randomized controlled ESA trials that reported mortality, conducted in both on-label and off-label ESA settings, were identified in a literature search conducted for a 2008 meeting between the Oncologic Drugs Advisory Committee to the Food and Drug Administration (FDA) and the companies that market ESAs in the US (Amgen Inc. and Centocor Ortho Biotech, which is a subsidiary of Johnson & Johnson) [27]. The meta-analysis of these data, along with three additional large meta-analyses, reported point estimates for mortality for which the confidence interval included 1 (95% confidence intervals [CI]: 0.99-1.18 [6], 0.90-1.45 [14], 0.95-1.12 [27], 0.98-1.18 [28]). Recently, two large meta-analyses (including a patient-level meta-analysis by the Cochrane Collaboration) of ESA use in all oncology settings reported 95% confidence intervals for mortality risk where the lower limit was equal to or slightly above 1 (95% CI: 1.00-1.12 [29],1.01-1.20 [30]). However, in the Cochrane Collaboration meta-analysis, the 95% confidence interval of the overall survival hazard ratio observed in 38 controlled ESA studies in the chemotherapy setting with 10,441 cancer patients overlapped 1 (1.04, 95% CI: 0.97 to 1.11) [29]. Unlike the previous 2006 Cochrane meta-analysis by Bohlius et al (2006) [6], this more recent meta-analysis from the Cochrane Collaboration also examined on-study deaths in the same set of 38 chemotherapy trials (hazard ratio = 1.10; 95% CI: 0.98 to 1.24) [29].

Because of the new environment surrounding ESA use in oncology and because varying results have been reported in meta-analyses of ESA oncology trials, additional trial data are needed to inform treatment decisions. Here we present an exploratory analysis examining hematological outcomes in CIA patients treated with darbepoetin alfa who were categorized in two baseline-Hb strata of < 10 g/dL or ≥10 g/dL. The purpose of this analysis was to provide insight into how changes in the approved ESA recommendations regarding the Hb level at which to initiate ESA therapy may impact patient care. The data for this analysis came from a pivotal, phase 3, double-blind, double-dummy, randomized, active-controlled darbepoetin alfa trial in CIA patients. This trial was chosen because it was a large randomized study that compared every-three-week (Q3W) and weekly (QW) darbepoetin alfa dosing schedules, which are the two schedules listed in the darbepoetin alfa product information [13,18]. Results previously published from this trial by Canon et al (2006) [11] indicated that 500 mcg Q3W darbepoetin alfa was non-inferior to 2.25 mcg/kg QW darbepoetin alfa in reducing the transfusion incidence from week 5 to end of the treatment period (EOTP) (the trial's primary endpoint) [11]. The Q3W and QW treatment arms also produced similar results for the secondary endpoints, which included the percentage of patients achieving a target Hb ≥11 g/dL from week 5 to EOTP, the change in Hb levels from baseline to EOTP, and safety outcomes [11]. In the present exploratory analysis from this trial, patients were analyzed according to baseline-Hb category (< 10 g/dL vs ≥10 g/dL); outcomes examined included the effect of Q3W or QW darbepoetin alfa on transfusion rates, Hb levels, and safety endpoints.

## Methods

### Patients and study design

The patient-eligibility criteria and study design for this phase 3, randomized, double-blind, active-controlled, double-dummy study are described in the primary report by Canon et al (2006) [11]. This trial was conducted in accordance with the Helsinki Declaration (ClinicalTrials.gov Identifier NCT00118638). An ethics committee approved the protocol at each study center, and patients provided informed consent before initiation of study procedures. Eligible patients (≥18 years of age) with CIA (Hb level < 11 g/dL within 24 hours prior to randomization), non-myeloid malignancy, at least 12 weeks of planned cytotoxic chemotherapy, and an Eastern Cooperative Oncology Group performance status of 0 to 2 were randomized 1:1 to receive 15 weeks of a fixed dose of 500 mcg Q3W darbepoetin alfa or a weight-based dose of 2.25 mcg/kg QW darbepoetin alfa [11]. Randomization was stratified by tumor type (lung or gynecologic vs others), screening Hb concentration (< 10 g/dL vs ≥10 g/dL), and European region (Western vs Central and Eastern). The dose of blinded study drug was withheld if a patient's Hb level was > 13 g/dL; after Hb levels decreased to ≤12 g/dL, study drug was reinstated at 60% of the previous dose. If Hb levels increased by ≥1 g/dL over a 14-day period (in the absence of a RBC transfusion during the previous 14 days), the dose of study drug was decreased to 60% of that used previously. Any additional dose reductions proceeded in 40% dose decrements. Transfusions were recommended, but not mandated, for patients with a Hb concentration ≤8 g/dL or > 8 g/dL with symptoms of anemia. All randomized patients who received at least one dose of study drug and remained in the study until at least day 29 were included in the analyses. Safety endpoints were evaluated according to treatment actually received in patients who received at least one dose of study drug.

The primary endpoint of the trial [11] was to examine if Q3W darbepoetin alfa was non-inferior to QW darbepoetin alfa for reducing transfusions from week 5 to EOTP (defined as the earlier of day 109 or end of study). This time period was chosen as ESA effects are often not apparent until the second month of ESA treatment [10]. This time period has been accepted by regulatory agencies as sufficient for drug approval. However, as some patients respond before that timeframe, a sensitivity analysis was also performed of transfusion incidence from week 1 to EOTP. Unadjusted Kaplan-Meier estimates were used to calculate a 2-sided 95% CI for the difference in the proportion of patients with at least one RBC transfusion from week 5 to EOTP for the Q3W vs QW regimen. Non-inferiority of the Q3W regimen was determined if the upper limit of the 95% CI for this difference was ≤12.5% (based on transfusion data from two placebo-controlled darbepoetin alfa trials) [8,10]. A sample size of 705 patients was originally determined to provide 95% power to demonstrate non-inferiority of the Q3W schedule compared with the QW schedule in regards to the transfusion rate from week 5 to EOTP.

### Endpoints and statistical methods for the exploratory analysis

For the exploratory analysis reported here, data from patients in the trial were analyzed by their baseline-Hb category: < 10 g/dL and ≥10 g/dL. Efficacy outcomes examined in the total population [11], and further summarized here, included the percentage of patients with RBC transfusions and the Hb profiles over time. In the < 10 g/dL baseline-Hb group, this exploratory analysis also assessed the incidence of patients who achieved a Hb level ≥10 g/dL and the time to first achievement of a Hb level ≥10 g/dL. Patients in both baseline-Hb groups who had a baseline Hb value and at least one post-baseline Hb value were examined for the percentage achieving a Hb level ≥11 g/dL, as well as for how long it took to achieve a Hb level ≥11 g/dL. There was one patient with Hb > 11 g/dL at both screening and baseline (ie, protocol violation), as well as a second patient who had Hb < 11 g/dL at screening which then rose to 11 g/dL at baseline (screening and baseline measures could be up to 4 days apart). These two patients were included in the study and dosed per the protocol, although they were not included in the analyses of patients achieving Hb ≥11 g/dL.

Safety endpoints examined in this exploratory analysis included the incidence of adverse events, the percentage of patients reaching a Hb level ≥12 g/dL or ≥13 g/dL at any time on study, and the proportion of patients with a rapid Hb increase of ≥1 g/dL in a 14-day window or ≥2 g/dL in a 28-day window.

Statistical analyses were performed using SAS statistical software (version 8.2, SAS Institute, Cary, NC). Descriptive statistics included frequencies with 95% CIs for categorical variables and means with standard deviations (SDs) for continuous variables. Unadjusted Kaplan-Meier estimates were used to analyze the incidence of transfusions and the proportion of patients achieving a specific Hb level. Hb concentrations measured within 28 days of a RBC or whole blood transfusion were excluded from the analysis. Analyses were not adjusted by stratification factors used at randomization. Adverse events were grouped by primary system organ class and by preferred term with the primary organ system class according to a MedDRA dictionary (version 7.0).

## Results

### Patient demographics and disease state

This phase 3 trial included 705 patients at 110 centers in 24 European countries [11]. In the present exploratory analysis, the 353 patients randomized to the 500 mcg Q3W darbepoetin arm in the primary study [11] were categorized into a baseline-Hb group of either < 10 g/dL (176 patients) or ≥10 g/dL (177 patients). The 352 patients randomized to the 2.25 mcg/kg QW darbepoetin arm were also categorized into a baseline-Hb group of either < 10 g/dL (175 patients) or ≥10 g/dL (177 patients). Thus, the < 10 g/dL baseline-Hb group contained 351 patients and the ≥10 g/dL baseline-Hb group contained 354 patients. The overall mean (SD) baseline Hb level in the < 10 g/dL baseline-Hb group was 9.05 g/dL (0.71), ranging from 5.9 to 9.9 g/dL, while the mean (SD) in the ≥10 g/dL baseline-Hb group was 10.48 g/dL (0.30), ranging from 10.0 to 11.8 g/dL (Table 1). Other than the baseline Hb level, the < 10 g/dL and ≥ 10 g/dL baseline-Hb groups had fairly similar demographics and baseline characteristics; these characteristics were also similar across the two darbepoetin dosing schedules (Table 1). Common tumor types in both the < 10 g/dL and ≥10 g/dL baseline-Hb groups included large intestine/colon, breast, and non-small cell lung cancer. Of note, slightly more patients in the ≥10 g/dL baseline-Hb group had colon cancer. Most patients analyzed had stage III/IV disease and were < 65 years of age. In patients receiving Q3W darbepoetin alfa, slightly more patients in the < 10 g/dL baseline-Hb group had stage III/IV disease. The majority of patients in this study had a performance status of 0 or 1 [11].

**Table 1:** Patient demographics and baseline disease state

	Baseline Hemoglobin < 10 g/dL			Baseline Hemoglobin ≥10 g/dL		
	
	Darbepoetin alfa 500 mcg Q3W N = 176	Darbepoetin alfa 2.25 mcg/kg QW N = 175	Total N = 351	Darbepoetin alfa 500 mcg Q3W N = 177	Darbepoetin alfa 2.25 mcg/kg QW N = 177	Total N = 354
Sex, n (%)						
Female	93 (53)	97 (55)	190 (54)	93 (53)	100 (56)	193 (55)
Age, years						
Median (Min, Max)	59 (20, 86)	61 (20, 83)	60 (20, 86)	61 (20, 85)	60 (21, 84)	60.5 (20, 85)
≥65, n (%)	59 (34)	65 (37)	124 (35)	65 (37)	65 (37)	130 (37)
≥75, n (%)	15 (9)	14 (8)	29 (8)	19 (11)	12 (7)	31 (9)
Tumor type, n (%)						
Large Intestine/Colon	30 (17)	16 (9)	46 (13)	35 (20)	35 (20)	70 (20)
Breast	23 (13)	27 (15)	50 (14)	34 (19)	28 (16)	62 (18)
NSCLC	17 (10)	15 (9)	32 (9)	17 (10)	17 (10)	34 (10)
Disease stage at diagnosis, n (%)						
I	13 (7)	10 (6)	23 (7)	15 (8)	8 (5)	23 (6)
II	25 (14)	38 (22)	63 (18)	40 (23)	37 (21)	77 (22)
III	53 (30)	49 (28)	102 (29)	42 (24)	44 (25)	86 (24)
IV	74 (42)	62 (35)	136 (39)	59 (33)	71 (40)	130 (37)
Other/Missing or Unknown	11 (6)	16 (9)	27 (8)	21 (12)	17 (10)	38 (11)
Prior chemotherapy, n (%)	169 (96)	153 (87)	322 (92)	162 (92)	154 (87)	316 (89)
Prior platinum chemotherapy, n (%)	68 (39)	59 (34)	127 (36)	59 (33)	63 (36)	122 (34)
Prior radiotherapy, n (%)	60 (34)	47 (27)	107 (30)	61 (34)	56 (32)	117 (33)
Prior erythropoietic therapy, n (%)^a^	21 (12)	21 (12)	42 (12)	18 (10)	13 (7)	31 (9)
Baseline hemoglobin, g/dL						
Mean (SD)	9.01 (0.78)	9.09 (0.64)	9.05 (0.71)	10.50 (0.29)	10.46 (0.30)	10.48 (0.30)
Median (Min, Max)	9.20 (5.9, 9.9)	9.20 (6.8, 9.9)	9.20 (5.9, 9.9)	10.50 (10.0, 10.9)	10.50 (10.0, 11.8)	10.50 (10.0, 11.8)

Q3W = every three weeks; QW = weekly; Min = minimum; Max = maximum; NSCLC = non-small cell lung cancer; SD = standard deviation

^a^Prior erythropoietic agents were not administered within 4 weeks of study day 1.

### Transfusions

Unadjusted Kaplan-Meier estimates were used to analyze the incidence of transfusions from week 5 to EOTP in the < 10 g/dL and ≥10 g/dL baseline-Hb groups (Table 2). In a sensitivity analysis, unadjusted Kaplan-Meier estimates were also used to analyze the incidence of transfusions from week 1 to EOTP in both baseline-Hb groups. The results indicated that the incidence of transfusions was lower in the ≥10 g/dL baseline-Hb group compared with the < 10 g/dL baseline-Hb group across both dosing schedules and for both time periods examined (from week 5 to EOTP and from week 1 to EOTP) (Table 2). In both baseline-Hb groups, the upper limit of the 95% CI for the difference in RBC transfusions (for both time periods examined) between the Q3W and QW groups was less than the pre-specified non-inferiority margin of 12.5%. These findings are consistent with results previously reported from this trial [11] indicating that darbepoetin alfa administered at 500 mcg Q3W is at least as effective as darbepoetin alfa administered at 2.25 mcg/kg QW for reducing transfusion requirements from week 5 to EOTP and from week 1 to EOTP.

**Table 2:** Incidence of transfusions

	Baseline Hemoglobin < 10 g/dL			Baseline Hemoglobin ≥10 g/dL		
	
	Darbepoetin alfa 500 mcg Q3W N = 176	Darbepoetin alfa 2.25 mcg/kg QW N = 175	Difference (Q3W-QW)	Darbepoetin alfa 500 mcg Q3W N = 177	Darbepoetin alfa 2.25 mcg/kg QW N = 177	Difference (Q3W-QW)
Week 5 to EOTP K-M percent (95% CI) [N]	36 (28 to 43) [164]	41 (33 to 49) [167]	-5.1 (-16.1 to 5.9)	14 (7 to 20) [171]	21 (14 to 29) [170]	-7.3(-17.3 to 2.8)
Week 1 to EOTP K-M percent (95% CI)	44 (37 to 52)	47 (39 to 54)	-2.3 (-13.2 to 8.6)	14 (8 to 21)	26 (18 to 33)	-11.3 (-21.4 to -1.2)

Q3W = every three weeks; QW = weekly; EOTP = end of the treatment period (defined as the earlier of day 109 or end of study); K-M = Kaplan-Meier

Of the 214 transfusions received by patients, 71% were in patients with baseline Hb < 10 g/dL. We also examined if the Hb level preceding the first transfusion was above or below the Hb threshold of 8 g/dL recommended (but not mandated) in the protocol (Table 3). For patients with baseline Hb < 10 g/dL, about half (48%) of first transfusions occurred at or below the protocol-specified threshold of 8 g/dL. For patients with baseline Hb ≥10 g/dL, 38% of the first transfusions occurred at or below the threshold of 8 g/dL.

**Table 3:** Number of first transfusions occurring at hemoglobin ≤8 g/dL or > 8 g/dL

		Baseline Hemoglobin < 10 g/dL		Baseline Hemoglobin ≥10 g/dL	
	
		Darbepoetin alfa 500 mcg Q3W	Darbepoetin alfa 2.25 mcg/kg QW	Darbepoetin alfa 500 mcg Q3W	Darbepoetin alfa 2.25 mcg/kg QW
First^a ^transfusion during week 5 to EOTP	Hb ≤8 g/dL, n (%)	30 (56)	29 (46)	12 (55)	12 (39)
	Hb > 8 g/dL, n (%)	24 (44)	34 (54)	10 (45)	19 (61)

First^a ^transfusion during week 1 to EOTP	Hb ≤8 g/dL, n (%)	42 (58)	30 (38)	9 (39)	14 (35)
	Hb > 8 g/dL, n (%)	30 (41)	48 (62)	14 (61)	24 (60)
	Hb missing, n (%)	1 (1)	0 (0)	0 (0)	2 (5)

Q3W = every three weeks; QW = weekly; EOTP = end of the treatment period (defined as the earlier of day 109 or end of study); Hb = hemoglobin; n = number of transfusion events. Note that as data described are for first transfusion only, n also equals the number of patients with at least one transfusion.

^a^The closest hemoglobin value on the day of transfusion or within 7 days prior was used.

### Hemoglobin endpoints

**Figure 1:**
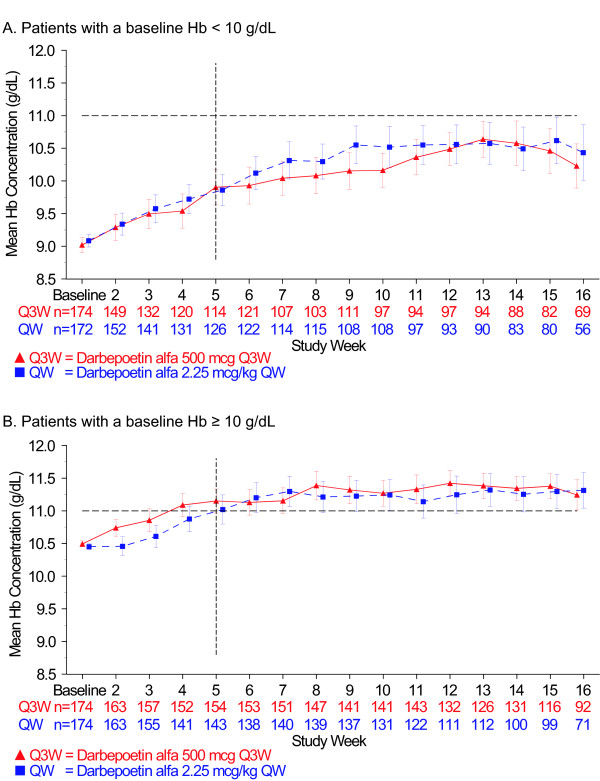
**Hemoglobin profile over the treatment period**. The mean hemoglobin (Hb) concentration (g/dL) is shown over time in weeks. The treatment period lasted through the earlier of day 109 or end of study. Error bars represent the 95% confidence intervals. The dotted, black horizontal line marks the 11 g/dL Hb level on the Y axis, and the dotted, black vertical line marks week 5 on the X axis. A. Patients with a baseline Hb level < 10 g/dL. B. Patients with a baseline Hb level ≥10 g/dL

In Figure 1, the mean (95% CI) Hb concentrations (g/dL) over the treatment period in weeks are shown for both baseline-Hb groups. By week 5, the ≥10 g/dL baseline-Hb group, but not the < 10 g/dL baseline-Hb group, achieved a mean Hb level ≥11 g/dL. The mean (SD) Hb concentration in the < 10 g/dL baseline-Hb group (Q3W vs QW dosing) was 9.90 g/dL (1.45) vs 9.86 g/dL (1.35) at week 5; 10.04 g/dL (1.37) vs 10.31 g/dL (1.54) at week 7; and 10.46 g/dL (1.57) vs 10.62 g/dL (1.6) at week 15. The mean (SD) Hb concentration in the ≥10 g/dL baseline-Hb group (Q3W vs QW dosing) was 11.15 g/dL (1.16) vs 11.02 g/dL (1.36) at week 5; 11.15 g/dL (1.24) vs 11.30 g/dL (1.39) at week 7; and 11.38 g/dL (1.02) vs 11.30 g/dL (1.30) at week 15. In both baseline-Hb groups, the mean Hb level remained under 12 g/dL throughout the entire treatment period (Figure 1).

**Figure 2:**
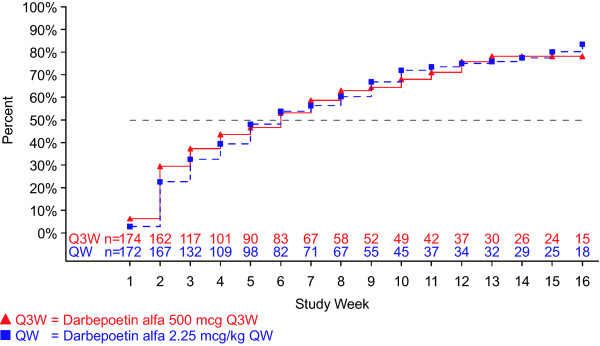
**Time to hemoglobin concentration ≥10 g/dL for patients with baseline hemoglobin < 10 g/dL**. Hemoglobin (Hb) measurements within 28 days of a red blood cell transfusion or whole blood transfusion were excluded. Patients not achieving a Hb level ≥10 g/dL between day 1 and end of the treatment period (EOTP, earlier of day 109 or end of study) were censored at their last Hb measurement prior to EOTP.

**Figure 3:**
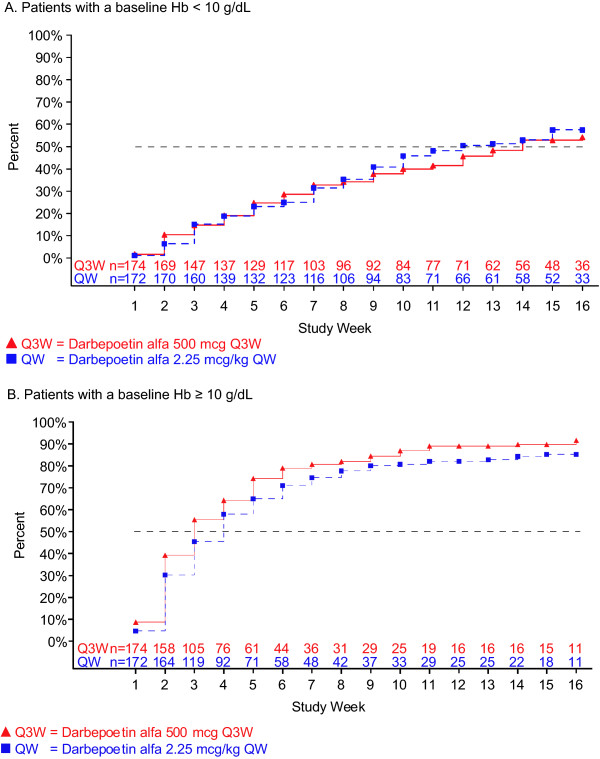
**Time to a hemoglobin concentration ≥11 g/dL**. Hemoglobin (Hb) measurements within 28 days of a red blood cell transfusion or whole blood transfusion were excluded. Patients not achieving a Hb level ≥11 g/dL between day 1 and end of the treatment period (EOTP, earlier of day 109 or end of study) were censored at their last Hb measurement prior to EOTP. Two patients in the ≥10 g/dL baseline-Hb group who had a baseline Hb level ≥11 g/dL were excluded from the analysis. A. Patients with a baseline Hb level < 10 g/dL. B. Patients with a baseline Hb level ≥10 g/dL.

We determined time to reach two Hb levels (10 g/dL and 11 g/dL) because of differing recommendations regarding ESA treatment present in the US and EU product information [16-19]. For patients in the < 10 g/dL baseline-Hb group, the unadjusted Kaplan-Meier estimated percentage (95% CI) of patients who achieved a Hb level ≥10 g/dL (Q3W vs QW dosing) was 43% (36 to 51) vs 48% (40 to 55) by week 5, 59% (51 to 66) vs 56% (48 to 64) by week 7, and 78% (70 to 84) vs 83% (76 to 89) by EOTP (Figure 2). The unadjusted Kaplan-Meier estimated percentage (95% CI) of patients in the < 10 g/dL baseline-Hb group who achieved a Hb level ≥10 g/dL (Q3W vs QW dosing) from week 5 to EOTP was 75% (68 to 82) vs 82% (75 to 89) and 78% (71 to 85) vs 83% (77 to 90) from week 1 to EOTP. The Kaplan-Meier median (95% CI) time to achieve a ≥10 g/dL Hb level (Q3W vs QW dosing) was 8 weeks (6 to 9) vs 7 weeks (6 to 9) from week 5 to EOTP and 6 weeks (4 to 7) vs 6 weeks (5 to 8) from week 1 to EOTP (Figure 2). Figure 3 shows the time for patients in both baseline-Hb groups to achieve a Hb level ≥11 g/dL. The unadjusted Kaplan-Meier percentage (95% CI) of patients (Q3W vs QW dosing) who achieved a Hb level ≥11 g/dL was 54% (46 to 62) vs 57% (49 to 65) in < 10 g/dL baseline-Hb group and 90% (85 to 94) vs 85% (79 to 90) in the ≥10 g/dL baseline-Hb group.

### Drug exposure and safety endpoints

The present exploratory analysis of the < 10 g/dL and ≥10 g/dL baseline-Hb groups examined drug exposure and safety outcomes. Table 4 summarizes drug exposure and the percentages of patients in both baseline-Hb groups who: 1) reached the Hb threshold of ≥12 g/dL or ≥13 g/dL, 2) had a dose withheld due to achievement of Hb > 13 g/dL, 3) had a rapid rate of Hb rise (≥1-g/dL Hb rise over a 14-day window or a ≥2-g/dL Hb rise over a 28-day window), or 4) had a dose reduction due to a rapid Hb rise (≥1-g/dL Hb rise in a 14-day window in the absence of a transfusion during the previous 14 days). Both the proportion of those who achieved a threshold Hb of ≥12 or ≥13 g/dL and the proportion of those who experienced a rapid rise in Hb were higher in the ≥10 g/dL baseline-Hb group.

**Table 4:** Incidence of hemoglobin levels ≥12 g/dL or ≥13 g/dL or rapid hemoglobin rises

	Baseline Hemoglobin < 10 g/dL		Baseline Hemoglobin ≥10 g/dL	
	
	Darbepoetin alfa 500 mcg Q3W N = 176	Darbepoetin alfa 2.25 mcg/kg QW N = 175	Darbepoetin alfa 500 mcg Q3W N = 177	Darbepoetin alfa 2.25 mcg/kg QW N = 177
Mean (SD) average weekly darbepoetin alfa dose, mcg/week^a^	134 (36)	115 (41)	125 (34)	112 (43)
Hemoglobin threshold of ≥12 g/dL achieved at any time during the study, n (%)^b^	44 (31)	49 (34)	109 (66)	99 (63)
Hemoglobin threshold of ≥13 g/dL achieved at any time during the study, n (%)^b^	20 (11)	24 (14)	56 (32)	60 (34)
Dose withheld due to achievement of > 13 g/dL hemoglobin, n (%)	4 (2)	22 (13)	12 (7)	57 (32)
Rapid rise in hemoglobin, n (%)				
≥1 g/dL in 14 days	99 (56)	103 (59)	133 (75)	119 (67)
≥2 g/dL in 28 days	48 (27)	54 (31)	70 (40)	64 (36)
Dose reduction due to rapid hemoglobin increase^c^, % (95% CI)	57 (49 to 64)	69 (61 to 75)	75 (67 to 81)	69 (62 to 76)

Q3W = every three weeks; QW = weekly

^a^Excluding withheld or missed doses.

^b^Hemoglobin measurements within 28 days of a RBC transfusion or whole blood transfusion were excluded.

^c^A rapid hemoglobin increase sufficient to trigger dose reduction was defined as ≥1 g/dL hemoglobin rise in a 14-day window in the absence of a transfusion during the previous 14 days.

The incidence of any adverse events (not necessarily treatment related) in the < 10 g/dL baseline-Hb group was 91% in both the Q3W and QW dosing groups. The incidence of any adverse event in the ≥10 g/dL baseline-Hb group was 85% in the Q3W dosing group and 88% in the QW dosing group. The most common adverse events in both baseline-Hb groups were nausea, vomiting, pyrexia, fatigue, and diarrhea. The incidence of serious adverse events (Q3W vs QW dosing) was 48% vs 42% in the < 10 g/dL baseline-Hb group and 31% vs 34% in the ≥10 g/dL baseline-Hb group. The most common serious adverse events in the < 10 g/dL baseline-Hb group (Q3W vs QW dosing) were pyrexia (6% of patients vs 6% of patients), febrile neutropenia (6% of patients vs 5% of patients), and anemia (3% of patients vs 5% of patients). The most common serious adverse events in the ≥10 g/dL baseline-Hb group (Q3W vs QW dosing) were pyrexia (4% of patients vs 5% of patients), deep-vein thrombosis (3% of patients vs 4% of patients), and anemia (2% of patients in each dosing group). Serious treatment-related adverse events (Q3W vs QW dosing) occurred in 3% vs 2% of patients in the < 10 g/dL baseline-Hb group and in 3% vs 4% of patients in the ≥10 g/dL baseline-Hb group. No anti-darbepoetin alfa antibodies were detected in any patients who received darbepoetin alfa in either baseline-Hb group [11].

In the < 10 g/dL and ≥10 g/dL baseline-Hb groups, safety analyses also examined the incidence of deaths on study, disease progression, and adverse events of historical interest, which include cardiovascular and thromboembolic events since there is a known risk of increased embolism/thrombosis with ESA use [6,30] (Table 5). Deaths over the whole study occurred in 11% of Q3W patients and in 15% of QW patients [11]; causes of death included infections, cardiac complications, and respiratory complications. Deaths were more frequent in the < 10 g/dL baseline-Hb group (Table 5). The incidences of most cardiovascular and thromboembolic events were similar in the two baseline-Hb groups except for a slightly increased incidence of arrhythmias in patients in the ≥10 g/dL baseline-Hb group who received QW darbepoetin alfa; there was also a slightly higher incidence of embolism/thrombosis events in the ≥10 g/dL baseline-Hb group compared with the < 10 g/dL baseline-Hb group.

**Table 5:** Incidence of adverse events of historical interest, death on-study, and disease progression^a^

	Baseline Hemoglobin < 10 g/dL		Baseline Hemoglobin ≥10 g/dL	
	
	Darbepoetin alfa 500 mcg Q3W N = 176	Darbepoetin alfa 2.25 mcg/kg QW N = 175	Darbepoetin alfa 500 mcg Q3W N = 177	Darbepoetin alfa 2.25 mcg/kg QW N = 177
Adverse events of historical interest, death on-study, and disease progression, n (%)	84 (48)	80 (46)	67 (38)	75 (42)
On-study deaths, n (%)	24 (14)	32 (18)	14 (8)	20 (11)
Disease progression, n (%)	42 (24)	37 (21)	29 (16)	36 (20)
Cardiovascular and thromboembolic events, n (%)	22 (12)	27 (15)	33 (19)	34 (19)
Arrhythmias	7 (4)	7 (4)	9 (5)	13 (7)
Cerebrovascular accident	0 (0)	2 (1)	0 (0)	1 (0.6)
Congestive heart failure	5 (3)	7 (4)	4 (2)	6 (3)
Myocardial infarction/Coronary artery disorders	2 (1)	3 (2)	3 (2)	0 (0)
Embolism/Thrombosis	11 (6)	11 (6)	19 (11)	17 (10)
Seizure, n (%)	1 (0.6)	0 (0)	0 (0)	1 (0.6)
Hypertension, n (%)	6 (3)	7 (4)	2 (1)	6 (3)
Pure red blood cell aplasia, n (%)	0 (0)	0 (0)	0 (0)	0 (0)
Immune system disorders, n (%)	0 (0)	1 (0.6)	1 (0.6)	2 (1)
Neoplasms benign, malignant, or unspecified (includes cysts/polyps), n (%)	44 (25)	33 (19)	14 (8)	26 (15)

Q3W = every three weeks; QW = weekly

^a^Includes all events within 28 days of the last dose of study drug (except serious adverse events, which were reported at any time after the first dose of study drug).

Of a total of 73 thromboembolic events reported during the study, 64% (47) occurred in patients with baseline Hb ≥10 g/dL. The most common thromboembolic events were: deep venous thrombosis (36%), general phlebitis/thrombosis (36%), and pulmonary embolism (18%). Thirty of the 73 events resulted in hospitalization, including 19 in patients with baseline Hb ≥10 g/dL. Twelve of these 19 hospitalizations occurred in patients on QW dosing, as compared with 7 on Q3W dosing. Of the 19 hospitalizations in patients with baseline Hb ≥10 g/dL, 10 were due to deep venous thrombosis and 6 to pulmonary embolism, with both events occurring with both dosing schedules. Two thromboembolic-related fatalities occurred, both in patients with pulmonary embolism. One of these patients had baseline Hb < 10 g/dL (Q3W dosing) and one had baseline Hb ≥10 g/dL (QW dosing).

## Discussion

The recent changes made globally to the ESA product information may affect how anemia is treated in cancer patients in both the EU and the US. The EU ESA product information states that ESA therapy be initiated in patients with baseline Hb ≤10 g/dL and that the target Hb range should be 10 to 12 g/dL [16,17]. In addition, the EU ESA product information also states that the decision to administer ESAs should be based on a benefit-risk assessment made with the participation of the individual patient that takes into account the specific clinical context (eg, the type of cancer and disease stage, the degree of anemia, life expectancy, and the environment in which the patient is being treated) and patient preference; in some clinical situations, blood transfusion should be the preferred treatment for anemia management in cancer patients [16,17]. The US ESA product information [18,19] states that ESAs be used to maintain the lowest possible Hb level while still avoiding transfusions. This exploratory analysis examined how the baseline Hb level (< 10 g/dL vs ≥10 g/dL) at which darbepoetin alfa administration was initiated in CIA patients affected response to darbepoetin alfa therapy. Overall, the results from this exploratory analysis indicated that darbepoetin alfa therapy corrected anemia in both baseline-Hb groups (though correction was faster in the ≥10 g/dL baseline-Hb group), irrespective of the dosing schedule (Q3W or QW). In addition, the incidence of most adverse events was generally similar between the two baseline-Hb groups.

In both baseline-Hb groups, Hb levels could be effectively raised with either the Q3W or QW darbepoetin alfa administration schedule to achieve Hb levels between 10 to 12 g/dL. In this analysis, initiating darbepoetin alfa therapy in patients with baseline Hb ≥10 g/dL (in both the Q3W and QW schedules) resulted in fewer transfusions compared with patients with a baseline Hb < 10 g/dL. Further, patients with ≥10 g/dL baseline Hb were more likely to achieve Hb levels between 11 and 12 g/dL (while still maintaining mean Hb levels below 12 g/dL) and to do so more quickly compared with patients with baseline Hb < 10 g/dL. However, since the original study was not designed to compare results in patients with baseline Hb < 10 g/dL with those seen in patients with a baseline Hb ≥10 g/dL, additional studies are needed to examine this point.

It should be noted that among patients with baseline Hb ≥10 g/dL, more achieved Hb levels ≥12 g/dL or ≥13 g/dL and more experienced a rapid rise in Hb than did patients with baseline Hb < 10 g/dL. Rises in Hb of that degree have also been observed in patients in placebo groups in controlled trials of darbepoetin alfa for CIA [8,10], which makes interpretation difficult. Additionally, a recent meta-analysis of six placebo-controlled darbepoetin alfa trials found no increased risk of death or disease progression in ESA-treated patients achieving higher Hb levels (>12 g/dL or 13 g/dL) or with a rapid rise in Hb [31]. However, it is possible that those patients who were more responsive to ESAs were generally healthier, as ESA response and improved survival could both reflect better heath status. Further, in a recent larger meta-analysis (53 trials), target Hb was not found to be associated with changes in mortality [29].

A particular concern is that in patients who start Q3W dosing, Hb levels will undergo transient excursions into concentrations that exceed the Hb level needed to avoid a transfusion or exceed the Hb range of 10 to 12 g/dL. In this study, the mean Hb concentrations remained below 12 g/dL for both baseline-Hb groups throughout the study, irrespective of the dosing schedule (Q3W or QW). With the dosing rules used in this study, the percentage of patients within each baseline-Hb group who reached a Hb concentration of ≥12 g/dL or ≥13 g/dL was comparable for both the Q3W and QW dosing groups, allaying concerns about Hb concentrations exceeding recommended levels with the Q3W schedule. Dose modifications in this study resulted predominantly from the dose reduction rule for patients with a Hb rise of ≥1 g/dL in a 14-day period. In the EU, the currently approved product information for darbepoetin alfa indicates similar dose reduction and stopping rules as those used in this trial (darbepoetin alfa administration was withheld in this trial at a Hb concentration > 13 g/dL and was reinstated at 60% of the previous dose after Hb levels decreased to 12 g/dL or less). Using titration of ESA doses may be the best method to achieve the lowest Hb dose to avoid transfusions or to maintain Hb levels in a range of 10 to 12 g/dL.

Though the safety profiles were generally similar in the two baseline-Hb groups, more deaths occurred in the < 10 g/dL baseline-Hb group. One possibility is that the patients with a lower baseline Hb level may have had a worse prognosis. Of interest, though the < 10 g/dL baseline-Hb group had more deaths, fewer thromboembolic events occurred in this group compared with the ≥10 g/dL baseline-Hb group.

An inherent limitation of this analysis was that it was not pre-specified (ie, it was exploratory). The original study was designed as a noninferiority efficacy study, and thus was not prospectively designed to assess safety. In addition, as with any post hoc analysis, performing multiple analyses can increase chance fluctuations in the outcomes. Therefore, these results cannot be considered as robust as those from a pre-specified analysis plan. Additional limitations arise from the original study design and heterogeneous patient population, both of which could confound results. Specifically, as this was a noninferiority study, there was no comparator placebo arm. Likewise, as this study was conducted at 110 medical centers in 24 countries, there could well be variations in transfusion practices from site to site or country to country. These differences were allowed by the protocol, which recommended, but did not mandate, transfusions for patients with Hb level ≤8 g/dL. For the patient population, other than having at least 12 additional weeks of planned cytotoxic chemotherapy, there were no limitations regarding chemotherapy type. Patients also had different stages of disease and a variety of tumor types, including lung, breast, colon, and hematologic. Patient populations with baseline Hb < 10 g/dL or ≥10 g/dL appeared generally well-balanced regarding stage and tumor type (Table 1); however, that does not rule out subtle differences that could affect interpretation. Lastly, the proportion of patients in each group who died could be a possible confounding factor. To determine what role this last factor might have, we examined survival curves and performed additional analyses using death as a competing risk (data not shown, methodology described in [32]). These analyses indicated that death or timing of death did not substantially affect the data.

Taken together, the data from this exploratory analysis indicate that patients with baseline Hb < 10 g/dL and ≥10 g/dL may benefit from ESA therapy. However, given our evolving understanding of possible long-term safety issues related to ESA use, including the effect of the Hb level at ESA initiation as well as target and/or achieved Hb levels, these results should be interpreted with caution. Ongoing long-term safety studies are expected to provide more clarity on these and other issues. Therefore, while these initial results contribute to the ongoing discussion of how to best use ESAs in patients with CIA, there are a variety of limitations of this exploratory analysis. Additional prospective studies that examine outcomes in patients treated with ESAs according to the current ESA product information will be of interest to clinical practice.

## Conclusion

Recent changes have been made globally to the ESA product information, including changes in the recommended baseline Hb level at which ESA therapy should be initiated in CIA patients. To examine how these changes may affect patient care, this exploratory analysis retrospectively examined outcomes in darbepoetin alfa-treated CIA patients who were categorized into baseline-Hb strata of < 10 g/dL or ≥10 g/dL at initiation of ESA therapy. In this analysis, darbepoetin alfa administered 500 mcg Q3W or 2.25 mcg/kg QW raised Hb levels in both baseline-Hb groups while still maintaining mean Hb levels below 12 g/dL as recommended by the EU ESA product information. However, patients with a baseline Hb level ≥10 g/dL had fewer transfusions and faster anemia correction compared with patients in the < 10 g/dL baseline-Hb group. Safety profiles were generally similar in both baseline-Hb groups except that the < 10 g/dL baseline-Hb group had a slightly higher incidence of deaths while the ≥10 g/dL baseline-Hb group had a slightly higher incidence of thromboembolic events and a higher incidence of patients reaching a Hb level ≥13 g/dL. Since this was a retrospective analysis, additional studies are warranted to examine how baseline Hb levels at initiation of ESA therapy affect hematological responses and safety outcomes.

## Competing interests

Johan Vansteenkiste has research support from the Amgen Chair in Supportive Cancer Care held at the University of Leuven.

Michael Hedenus has been and is a member of temporary advisory boards for Amgen Sweden and has also received minor honoraria from Amgen Inc.

Pere Gascon has received honoraria from Amgen Inc. for lectureships.

Carsten Bokemeyer has received honoraria from Amgen Inc. for seminars and has been on advisory boards for Amgen Inc., Centocor Ortho Biotech, and Roche. Carsten Bokemeyer also declares that Amgen Inc. has funded two clinical studies on darbepoetin alfa in oncology at his institutional department.

Heinz Ludwig has participated in advisory boards for Amgen Inc., Centocor Ortho Biotech, and Roche.

Jan Vermorken declares no competing interests.

Lisa Hamilton is an employee of Amgen Inc. and owns Amgen Inc. stock.

Ken Bridges is an employee of Amgen Inc. and owns Amgen Inc. stock.

Beatriz Pujol is an employee of Amgen Inc. (Medical Director International Development) and owns Amgen Inc. stock.

## Authors' contributions

All authors read and approved the final manuscript. JV participated in the study conception and design; was involved in collecting, analyzing, and interpreting the data; and helped draft the manuscript. MH was involved in collecting the data and helped draft the manuscript. PG was involved in analyzing and interpreting the data and helped draft the manuscript. CB participated in the study conception and design; was involved in analyzing and interpreting the data; and helped draft the manuscript. HL was involved in analyzing and interpreting the data and helped draft the manuscript. JV was involved in analyzing and interpreting the data and helped draft the manuscript. LH participated in the study conception and design; was involved in analyzing and interpreting the data; and helped draft the manuscript. KB was involved in analyzing and interpreting the data and helped draft the manuscript. BP was involved in collecting, analyzing, and interpreting the data as well as helped to draft the manuscript.

## Pre-publication history

The pre-publication history for this paper can be accessed here:

http://www.biomedcentral.com/1471-2407/9/311/prepub

## References

[B1] Groopman JE Itri LM Chemotherapy-induced anemia in adults: incidence and treatment J Natl Cancer Inst 1999 91 19 1616 1634 10.1093/jnci/91.19.1616 10511589

[B2] Cella D Dobrez D Glaspy J Control of cancer-related anemia with erythropoietic agents: a review of evidence for improved quality of life and clinical outcomes Ann Oncol 2003 14 4 511 519 10.1093/annonc/mdg167 12649095

[B3] Barbara JA The rationale for pathogen-inactivation treatment of blood components Int J Hematol 2004 80 4 311 316 10.1532/IJH97.04120 15615254

[B4] Looney MR Gropper MA Matthay MA Transfusion-related acute lung injury: a review Chest 2004 126 1 249 258 10.1378/chest.126.1.249 15249468

[B5] Khorana AA Francis CW Blumberg N Culakova E Refaai MA Lyman GH Blood transfusions, thrombosis, and mortality in hospitalized patients with cancer Arch Intern Med 2008 168 21 2377 2381 10.1001/archinte.168.21.2377 19029504 PMC2775132

[B6] Bohlius J Wilson J Seidenfeld J Piper M Schwarzer G Sandercock J Trelle S Weingart O Bayliss S Djulbegovic B Recombinant human erythropoietins and cancer patients: updated meta-analysis of 57 studies including 9353 patients J Natl Cancer Inst 2006 98 10 708 714 16705125 10.1093/jnci/djj189

[B7] Gabrilove JL Cleeland CS Livingston RB Sarokhan B Winer E Einhorn LH Clinical evaluation of once-weekly dosing of epoetin alfa in chemotherapy patients: improvements in hemoglobin and quality of life are similar to three-times-weekly dosing J Clin Oncol 2001 19 11 2875 2882 11387360 10.1200/JCO.2001.19.11.2875

[B8] Hedenus M Adriansson M San Miguel J Kramer MH Schipperus MR Juvonen E Taylor K Belch A Altes A Martinelli G Efficacy and safety of darbepoetin alfa in anaemic patients with lymphoproliferative malignancies: a randomized, double-blind, placebo-controlled study Br J Haematol 2003 122 3 394 403 10.1046/j.1365-2141.2003.04448.x 12877666

[B9] Littlewood TJ Bajetta E Nortier JW Vercammen E Rapoport B Effects of epoetin alfa on hematologic parameters and quality of life in cancer patients receiving nonplatinum chemotherapy: results of a randomized, double-blind, placebo-controlled trial J Clin Oncol 2001 19 11 2865 2874 11387359 10.1200/JCO.2001.19.11.2865

[B10] Vansteenkiste J Pirker R Massuti B Barata F Font A Fiegl M Siena S Gateley J Tomita D Colowick AB Double-blind, placebo-controlled, randomized phase III trial of darbepoetin alfa in lung cancer patients receiving chemotherapy J Natl Cancer Inst 2002 94 16 1211 1220 12189224 10.1093/jnci/94.16.1211

[B11] Canon JL Vansteenkiste J Bodoky G Mateos MV Bastit L Ferreira I Rossi G Amado RG Randomized, double-blind, active-controlled trial of every-3-week darbepoetin alfa for the treatment of chemotherapy-induced anemia J Natl Cancer Inst 2006 98 4 273 284 16478746 10.1093/jnci/djj053

[B12] Pirker R Ramlau RA Schuette W Zatloukal P Ferreira I Lillie T Vansteenkiste JF Safety and efficacy of darbepoetin alfa in previously untreated extensive-stage small-cell lung cancer treated with platinum plus etoposide J Clin Oncol 2008 26 14 2342 2349 10.1200/JCO.2007.15.0748 18467726

[B13] Aranesp^® ^(Darbepoetin alfa) Package Insert. Amgen Inc., Breda, The Netherlands 2008

[B14] Ross SD Allen IE Henry DH Seaman C Sercus B Goodnough LT Clinical benefits and risks associated with epoetin and darbepoetin in patients with chemotherapy-induced anemia: a systematic review of the literature Clin Ther 2006 28 6 801 831 10.1016/j.clinthera.2006.06.003 16860166

[B15] Bokemeyer C Aapro MS Courdi A Foubert J Link H Osterborg A Repetto L Soubeyran P EORTC guidelines for the use of erythropoietic proteins in anaemic patients with cancer: 2006 update Eur J Cancer 2007 43 2 258 270 10.1016/j.ejca.2006.10.014 17182241

[B16] European public assessment reports for authorised medicinal products for human use. European Medicines Agency website http://www.emea.europa.eu/htms/human/epar/a.htm

[B17] electronic Medicines Compendium (eMC) website http://emc.medicines.org.uk

[B18] Aranesp^® ^(Darbepoetin alfa) Package Insert 2009 Amgen Inc., Thousand Oaks, CA

[B19] Procrit^® ^(Epoetin alfa) Package Insert 2009 Centocor Ortho Biotech Products, L.P., Raritan, NJ

[B20] Amgen announces interim results of Aranesp^® ^"PREPARE" study in breast cancer patients [Amgen press release]. Nov 30, 2007 http://wwwext.amgen.com/media/media_pr_detail.jsp?year=2007&releaseID=1083091

[B21] Henke M Laszig R Rube C Schafer U Haase KD Schilcher B Mose S Beer KT Burger U Dougherty C Erythropoietin to treat head and neck cancer patients with anaemia undergoing radiotherapy: randomised, double-blind, placebo-controlled trial Lancet 2003 362 9392 1255 1260 10.1016/S0140-6736(03)14567-9 14575968

[B22] Leyland-Jones B Semiglazov V Pawlicki M Pienkowski T Tjulandin S Manikhas G Makhson A Roth A Dodwell D Baselga J Maintaining normal hemoglobin levels with epoetin alfa in mainly nonanemic patients with metastatic breast cancer receiving first-line chemotherapy: a survival study J Clin Oncol 2005 23 25 5960 5972 10.1200/JCO.2005.06.150 16087945

[B23] Overgaard J Hoff C Sand Hansen H Specht L Overgaard M Grau C Andersen E Johansen J Andersen L Evensen J Randomized study of the importance of Novel Erythropoiesis Stimulating Protein (Aranesp^®^) for the effect of radiotherapy in patients with primary squamous cell carcinoma of the head and neck (HNSCC)- the Danish Head and Neck Cancer Group DAHANCA 10 randomized trial [abstract 6LB] Eur J Cancer 2007 4 7

[B24] Smith RE Jr Aapro MS Ludwig H Pinter T Smakal M Ciuleanu TE Chen L Lillie T Glaspy JA Darbepoetin alfa for the treatment of anemia in patients with active cancer not receiving chemotherapy or radiotherapy: results of a phase III, multicenter, randomized, double-blind, placebo-controlled study J Clin Oncol 2008 26 1040 1050 10.1200/JCO.2007.14.2885 18227526

[B25] Thomas G Ali S Hoebers FJ Darcy KM Rodgers WH Patel M Abulafia O Lucci JA 3rd Begg AC Phase III trial to evaluate the efficacy of maintaining hemoglobin levels above 12.0 g/dL with erythropoietin vs above 10.0 g/dL without erythropoietin in anemic patients receiving concurrent radiation and cisplatin for cervical cancer Gynecol Oncol 2008 108 2 317 325 10.1016/j.ygyno.2007.10.011 18037478 PMC2350198

[B26] Wright JR Ung YC Julian JA Pritchard KI Whelan TJ Smith C Szechtman B Roa W Mulroy L Rudinskas L Randomized, double-blind, placebo-controlled trial of erythropoietin in non-small-cell lung cancer with disease-related anemia J Clin Oncol 2007 25 9 1027 1032 10.1200/JCO.2006.07.1514 17312332

[B27] Amgen Inc. in collaboration with Johnson & Johnson Pharmaceutical Research and Development, L.L.C.Background Information. For The Oncologic Drugs Advisory Comittee (ODAC) Meeting 13 March 2008 http://www.fda.gov/ohrms/dockets/ac/08/briefing/2008-4345b2-00-FDA-index.htm

[B28] Seidenfeld J Piper M Bohlius J Weingart O Trelle S Engert A Skoetz N Schwarzer G Wilson J Brunskill S Comparative effectiveness of epoetin and darbepoetin for managing anemia in patients undergoing cancer treatment. Comparative Effectiveness Review No. 3. (Prepared by Blue Cross and Blue Shield Association Technology Evaluation Center Evidence-based Practice Center under Contract No. 290-02-0026) 2006 Rockville, MD: Agency for Healthcare Research and Quality http://effectivehealthcare.ahrq.gov/repFiles/EPO%20Final.pdf 20704045

[B29] Bohlius J Schmidlin K Brillant C Schwarzer G Trelle S Seidenfeld J Zwahlen M Clarke M Weingart O Kluge S Recombinant human erythropoiesis-stimulating agents and mortality in patients with cancer: a meta-analysis of randomised trials Lancet 2009 373 9674 1532 1542 10.1016/S0140-6736(09)60502-X 19410717

[B30] Bennett CL Silver SM Djulbegovic B Samaras AT Blau CA Gleason KJ Barnato SE Elverman KM Courtney DM McKoy JM Venous thromboembolism and mortality associated with recombinant erythropoietin and darbepoetin administration for the treatment of cancer-associated anemia JAMA 2008 299 8 914 924 10.1001/jama.299.8.914 18314434

[B31] Ludwig H Crawford J Osterborg A Vansteenkiste J Henry DH Fleishman A Bridges K Glaspy JA Pooled Analysis of Individual Patient-Level Data From All Randomized, Double-Blind, Placebo-Controlled Trials of Darbepoetin Alfa in the Treatment of Patients With Chemotherapy-Induced Anemia J Clin Oncol 2009 27 17 2838 2847 10.1200/JCO.2008.19.1130 19380447

[B32] Satagopan JM Ben-Porat L Berwick M Robson M Kutler D Auerbach AD A note on competing risks in survival data analysis Br J Cancer 2004 91 7 1229 35 10.1038/sj.bjc.6602102 15305188 PMC2410013

